# Exposure of neonatal mice to Sevoflurane may induce male germ cell apoptosis in testicular tissue after puberty

**Published:** 2017-08

**Authors:** Maryam Nezhad Sistani, Anahid Maleki, Maryam Salimi, Marefat Ghaffari Novin, Hamid Nazarian

**Affiliations:** 1 *Department of Biology and Anatomical Sciences, Faculty of Medicine, Shahid Beheshti University of Medical Sciences, Tehran, Iran.*; 2 *Department of Anesthesiology, Faculty of Medicine, Tehran University of Medical Sciences, Tehran, Iran.*

**Keywords:** Spermatogonial cell, Prepubertal, Germ cells

## Abstract

**Background:**

common use of sevoflurane in congenital defects during repeated surgeries may have detrimental effects on spermatogenesis after puberty.

**Objective:**

This study investigated sevoflurane effects on spermatogenesis process in male mature mice after exposure in prepubertal time.

**Materials and Methods:**

24 neonatal NMRI male mice were randomly classified in three groups. Experimental 1 and 2 groups (exposure to 1 minimum alveolar concentration (MAC) and 2 MAC sevoflurane, respectively in 2 lit/min oxygen (O_2_) for 7 days (30 min, daily) and control. All groups were sacrificed after 2 months. Histological assessment, immunohistochemistry and apoptosis process was done. Bax and Bcl_2_ expression was evaluated in the testicular tissue by real time Poly Chain Reaction**.**

**Results:**

Our results showed that the integrity of testicular tissue was preserved in both experimental groups. Count of spermatogonial cells had significant decrease in group 2 compared to others. The rate of apoptosis in spermatogonial cells was 15±3% and 9±2% in the group 2 and 1, respectively. Also, Bax/Bcl_2_ ratio was 0.2615, 1.0070 and 9.3657 in control, experimental group 1 and 2, respectively. This result was significant (p≤0.002) between groups 2 with other groups.

**Conclusion:**

Continuous exposure of 2 MAC sevoflurane in 2 lit/min O_2_ simultaneous during prepubertal may create more testicular tissue damage in terms of cellular and molecular function compared to continuous exposure to lower level of sevoflurane by increase in ratio of Bax/Bcl_2_ and apoptosis in germ cells after puberty.

## Introduction

Sevoflurane or fluoromethyl used for initiation and maintenance of general anesthesia. In modern anesthesiology, isofluorane and halothane are replaced by sevoflurane (1). The minimum alveolar concentration (MAC) of sevoflurane has been declared between 1.71-3.5% (2, 3). The pharmacodynamic outcomes of sevoflurane on the different organ systems are similar to other halogenated ethers (4). Sevoflurane decompose by the liver to generate some ions like inorganic fluoride (5, 6). 

In humans, up to 5% of the administered dose of sevoflurane usually is metabolized by the 2E1 isoform of cytochrome P450 (7-10). Experimental studies have revealed that P450 can be induced by phenobarbitone, isoniazid and ethanol, causing to elevation of serum inorganic fluoride level and excretion of fluoride ions (11-14). 

Gene expression profiling of the sperm cells from fluoride-exposed mice have shown that expression of 34 genes were increased and 63 genes were decreased, most of which are involved in the signal transduction, phosphorylation of amino acid, reactive oxygen specious, mitosis, spermatogenesis, and apoptosis (15). The human population is being exposed to many factors contributing to growing infertility. Fluoride and aluminium ions are two of the potential agents which affect fertility rate (16). Increasing exposure to ﬂuorides might have a negative effect on both female and male fertility, including spermatogenesis, sperm parameters, and sperm fertilization potential (17-22).

During the last decades, fluoride produced by sevoflurane was established to be a potent inducer of cell death in many cell types and experimental animal models. It should be noted that important progress has been promoted towards understanding the molecular mechanisms of fluoride ion. This ion progresses all known intracellular signaling pathways such as G protein-dependent signaling cascade, caspases pathway, and mitochondria- and death receptors-linked mechanisms, as well as triggering a range of metabolic and transcription changes, including the expression of some apoptosis-related genes ultimately leading to cell death (16). Although many proteins are involved in the development of fluoride-induced apoptosis have been identified to date, most of the pathways and the intracellular signaling process and time-dependent chains of events are still unknown. However, due to the common use of sevoflurane during repeated surgeries within a limited time, it is likely to have detrimental effects on spermatogenesis after puberty. 

The main goal of this study was to evaluate the effect of sevoflurane during neonatal life period on maturity and spermatogenesis in mature male mice.

## Materials and methods

**Chemicals**

Sevoflurane was purchased from Abbott, UK, Ham’s F10 was purchased from Sigma-Aldrich Co (USA,SaintLouis,MO), QIAzol was purchased from Qiagene, Germany, cDNA synthesis kit was purchased from Thermo Scientific, Fermentas, Waltham, MA, USA, In Situ Cell Death Detection Kit was bought from POD; Germany.

**Animals**

In a randomized experimental study, several pregnant female NMRI mice were bought from the Pasteur Institute (Tehran, Iran). Twenty four neonatal male mice were included in this study. 48 hr after birth, the neonatal male mice were randomly divided into three groups included experimental 1 and 2 groups (exposure to 1 MAC and 2 MAC sevoflurane respectively which was in 2 lit/min O_2_ simultaneous for seven consecutive days (30 min, daily) and control (exposure to 2 lit/min O_2_ only corresponding to experimental groups). after the last exposure to sevoflurane, they were housed in a standard laboratory conditions (12 hr light/dark at 22^o^C) with free access to water and food for two months (17). The 50×50×50 cm poly ethylene chamber was used as an exposure chamber. Inlet and outlet was in left and right side of chamber for oxygen and sevoflurane to enter and exit.

**Histopathological examinations of samples**

For histological studies, the testis and epididymis were fixed in 10% formalin, dehydrated in ethanol and embedded in paraffin. Sample sections (5 µm) were mounted on glass slides coated with Mayer’s albumin and dried overnight. Then, the sections were deparaffinzed with xylene, and rehydrated with alcohol and water. The rehydrated sections were stained using H&E, and were mounted with DPX mounting media and examined under the microscope (Olympus BX51 Tokyo, Japan). The testis and epididymis sections from each animal were evaluated for morphological changes. 

**Spermatogonial and Sertoli cells population in somniferous tubule**

For characterization of spermatogonial and Sertoli cells, immunohistochemistry was done for *Plzf *and *Oct4* as germ cell markers and *Vimentin* as a Sertoli cell marker. After sectioning of parrafined tissue, the samples were fixed with 4% paraformaldehyde (Sigma-Aldrich) for 20 minutes at room temperature, washed with PBS, incubated with HCl (2N) for 30 min at room temperature for antigen retrieval, washed with burate buffer twice, permeabilized with 3% Triton X-100 in PBS, and nonspecific reactions were blocked with 10% normal goat serum. Samples were maintained for 16 hour with *Plzf*, *Oct4* & *Vimentin* antibodies (Abcam, UK) diluted 1:100 in PBS. three PBS washes were done and then, the preparations were incubated for another 30 minutes with a 1:100 dilution of secondary FITC anti-rabbit IgG antibody (Abcam, UK) and finally were dried and mounted. 

**Measuring of apoptotic gene expression**

Total RNA, was isolated by QIAsoul kit (QIAgene). Genomic contamination was removed with DNase I treatment kit (Fermentase, Lithuania) based on the manufacturer‘s protocol. UV spectrophotometer was used for reading of RNA concentrations (Eppendorff, Germany). 1000 ng DNase-treated RNA samples were used to cDNAs synthesis from by a cDNA synthesis kit (Fermentase, Lithuania) using oligo (dT) primers. For PCR reactions, using Allele ID software, the desired primers, were designed (Bax, Bcl_2_ and Gapdh genes as housekeeping and normalizer gene) (Table I).

A final volume reaction was 25 μl, using 1000ng of cDNA was applied to make a PCR reaction. The real time-PCR cycling time were 95^o^C for 15 sec, 60^o^C for 30 sec, 72^o^C for 30 sec, followed by 40 cycles. The temperature melting curve program was considered between 60^o^C and 95^o^C. Efficiency was determined using a standard curve (the logarithmic dilution series of testis cDNA). the expression of two genes from apoptotic family (*Bax, Bcl*_2_) in each group was evaluated and each reaction was repeated three times. β-actin Gapdh gene as a reference gene was used. Expression of genes was calculated base on Pfaffl formula (the following method). 

**Measuring of sperm parameters**

For sperm analyze, epididymis of each mouse was removed and after placement in the 1 ml Ham’s F10 + 5% BSA,it was minced and incubated for 30 min at 37^o^C and 6% CO_2_ until sperm cells were swimmed into culture medium. One drop of the sperm suspension was put on a slide for light microscope observation. The sperm motility was investigated under the ×400 magnification. Neubaur haemocytometer slide was used for sperm count. The smear of the sperm suspension was stained with Diff-Quick for estimating the number of sperm with normal morphology. 

A total of 200 normal and abnormal sperm cells were counted under the light microscopy ×1000. Sperm viability was determined by Eosin-Nigrosin dye to estimate permeability of sperm membrane. Stained cells were counted as dead cells and unstained cells were considered as vital cells. 

**Quantification of apoptotic testicular cells by TUNEL assay**

TUNEL staining was used in order to estimate the number of apoptotic cells in testicular tissue. In-Situ-Cell Death Detection Kit was used for this purpose. The paraffin-embedded testicular tissue was deparaffinized, rehydrated and washed in PBS, incubated for 15-30 minutes at 37^o^C with proteinase K, permeabilized with permeabilization solution (0.1% Triton x-100, 0.1% sodium citrate), incubated in TUNEL solution (450 μl of label solution added with 50μl of enzyme solution) at 37^o^C for 1 hr. Finally the samples were washed with PBS, and observed under a fluorescent microscope (×400). The total number of apoptotic cells was counted in 5 seminiferous tubules sections in each group. 

**Ethical consideration**

The current study was conducted under the protocol approved by the animal experimentation committee of medical sciences faculty in Tehran University of medical sciences (IR.TUMS.REC.1394.13). 

**Statistical analysis**

Data were presented as mean±SE (standard error) and were investigated using One-way repeated measure analysis of variance (ANOVA) followed by Tukey’s post hoc test. P<0.05 were considered statistically significant.

## Results

**Histopathological examinations of samples**

**Figure 1:**
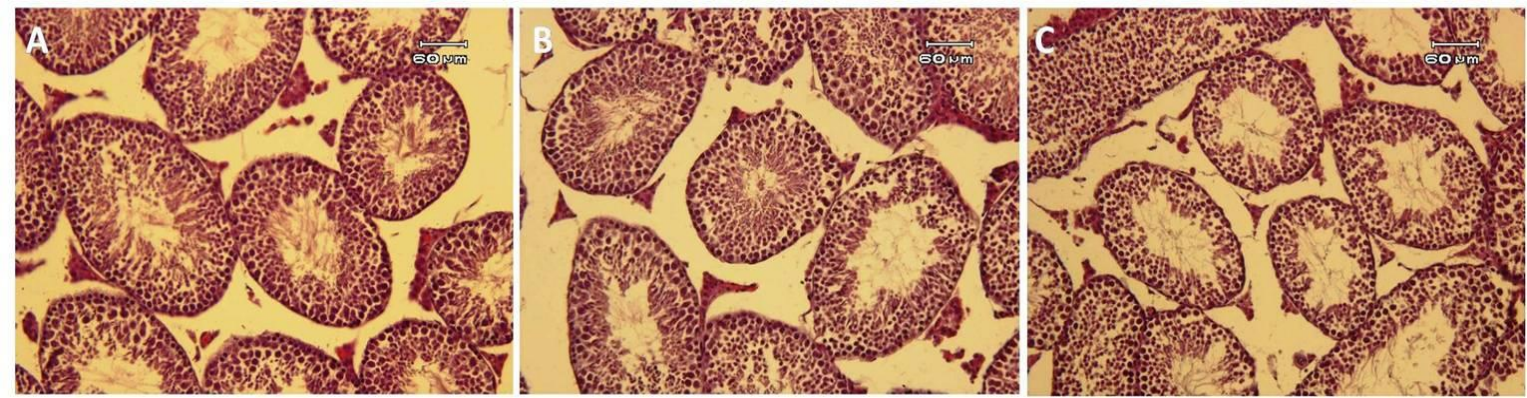
Histological assessment of testicular tissue in mice: in the control group (A), experimental group 1 (B) and experimental group 2 (C); H & E staining

The tissue samples in all experimental groups showed normal integrity of seminiferous tubule followed by all types of germ line cells. The seminiferous epithelium was characterized by the presence of Sertoli cells, spermatogonia cells, numerous spermatocytes, spermatids, and spermatozoa. There were significantly more Leydig cells in the control group than in two experimental groups. Differences between the types of exposure in the experimental groups became histologically apparent in the number of sperm in the central lumen and the patency of the seminiferous tubules internal space. There were few germ cells in the experimental group 2 in comparison with the other experimental groups (Figure 1C). 

The seminiferous tubules of the experimental 1 and control were found to contain spermatozoa and spermatogenesis was fully established (including elongated spermatids) in contrast to the experimental group 2 (Figure 1A & B). However, poor interaction between the Sertoli cell and germ cell was seen. Seminiferous tubules and epididymides in mice in the control group were regular. Normal interaction between the Sertoli cell and germ cell was seen in mice in the control group. The space between the seminiferous tubules contained some Leydig cells and internal lumen was full of spermatozoa as well as cauda of epididymis (Figure 1A).

**Identification of spermatogonial stem cell and Sertoli cells in somniferous tubule**

**Figure 2:**
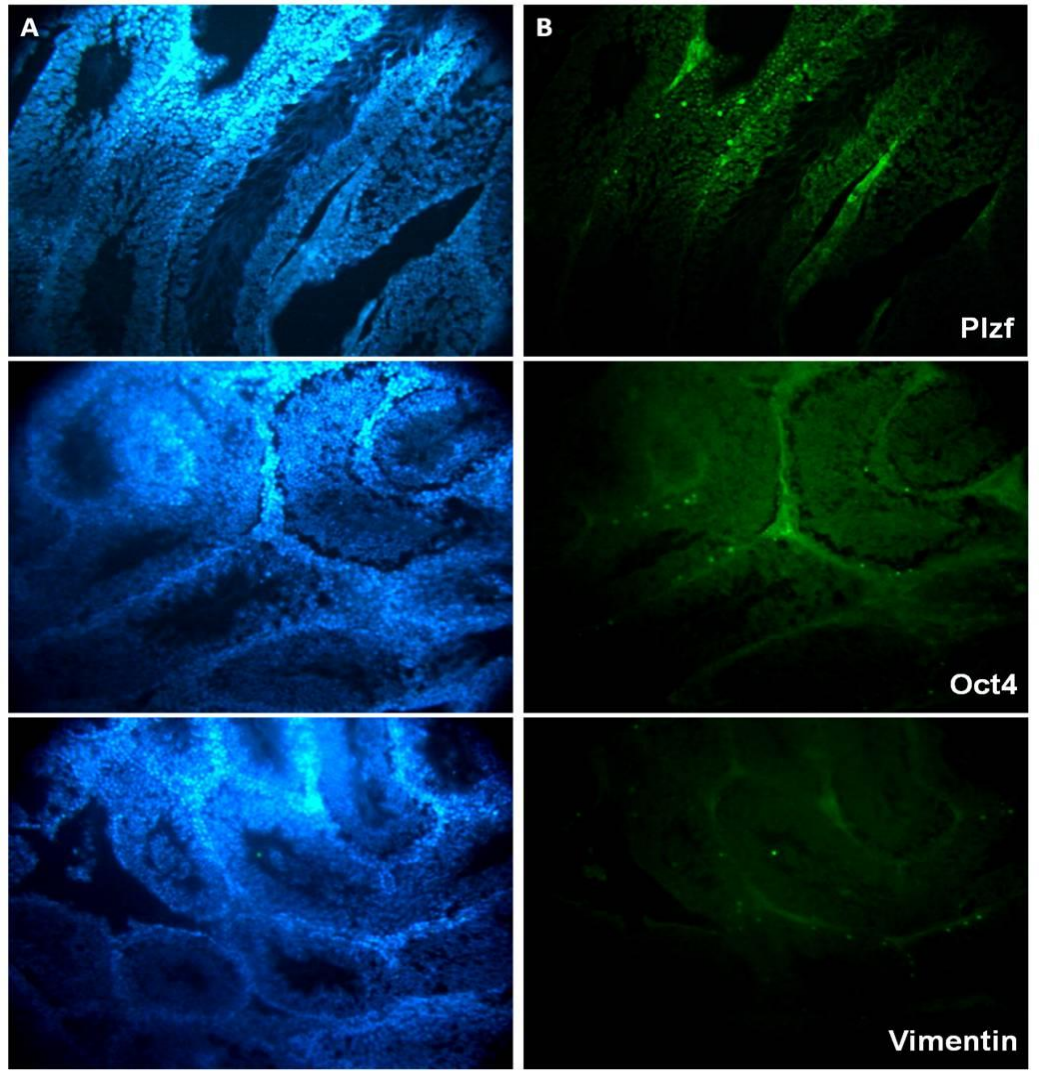
Immunohistochemistry assessment of testicular tissue in mice: Nuclei stain by DAPI (A), positive reaction with specific spermatogonial cell [Plzf], spermatogonial stem cells [Oct4] and Sertoli cells [Vimentin]. (B) Secondary antibody conjugated with FITC. Magnification: ×200

Spermatogonial stem cells, spermatogonial and Sertoli cells were characterized in somniferous tubule in all experimental groups. Based on our results, population of spermatogonial stem cells did not have any decrease as well as Sertoli cells. Also, spermatogonial cells population were decreased in experimental group 2 in comparison with other groups. However, it was no significant difference between two experimental groups with the controls (Figure 2).

**Sperm viability**

Significant increase in the number of dead sperm cells in the experimental group 1 and 2 with separate exposure to 1 and 2 MAC sevoflurane were observed in comparison with the control group (p<0.01). The sperm viability in the control group was 94.2+1.92%. However, the sperm viability in the experimental groups 1 and 2 was the same, but was lower than that of the control group (Table II).

**Sperm morphology**

Any kind of abnormality in sperm appearance, head, tail, and remaining cytoplasmic residues was considered as the morphological abnormalities. There was a decrease in the number of spermatozoa with normal morphology in experimental groups 1 and 2 in comparison with the control group (Table II). 

**Sperm motility**

The mean number of motile spermatozoa in the experimental groups 1 and 2 was 21+17.1464% & 38.8+9.3648% more similar to each other and no significant difference was observed between the two groups. Statistically significant difference was reported in the mean number of motile sperm cells in all experimental groups in comparison with the control group (p<0.04).

**Mean sperm count**

The mean number of spermatozoa in mice in the experimental group 1 and 2 were 9.036+0.89895 & 10.036+0.2275 (×10^6^), respectively. As shown in Table 2, there was no significant decrease in the mean number of spermatozoa in mice in the experimental groups with separate exposure to 1 and 2 MAC sevoflurane in comparison with the control group. 

**Apoptotic genes expression analysis**

**Figure 3:**
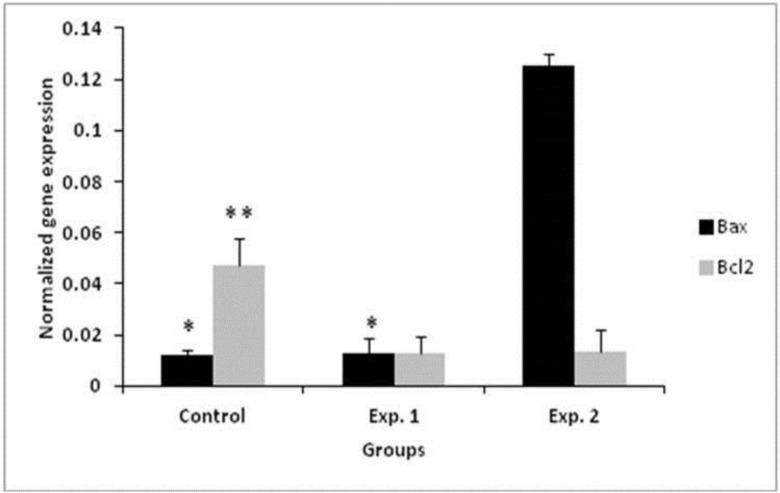
Real time PCR results for assessment of the apoptotic gene expression in the control and experimental groups.

Expression of both genes was normalized relative to the housekeeping gene in each group (Figure 1). There were significant differences between expressions of both genes in then experimental group 2 compared to the control group (p<0.045). The results showed that *Bax* expression in control, experimental group 1 and 2 was 0.01229±0.001, 0.01279+0.01, 0.41252±0.001; respectively (Figure 3). Also, *Bcl*_2_ expression in control, experimental group 1 & 2 was 0.04699±0.01, 0.01270+0.001, 0.01337±0.01; respectively.

**Apoptotic positive cells assessment**

The results showed that there was a significant difference between the experimental group 2 compared to other groups (p<0.03). 

**Figure 4:**
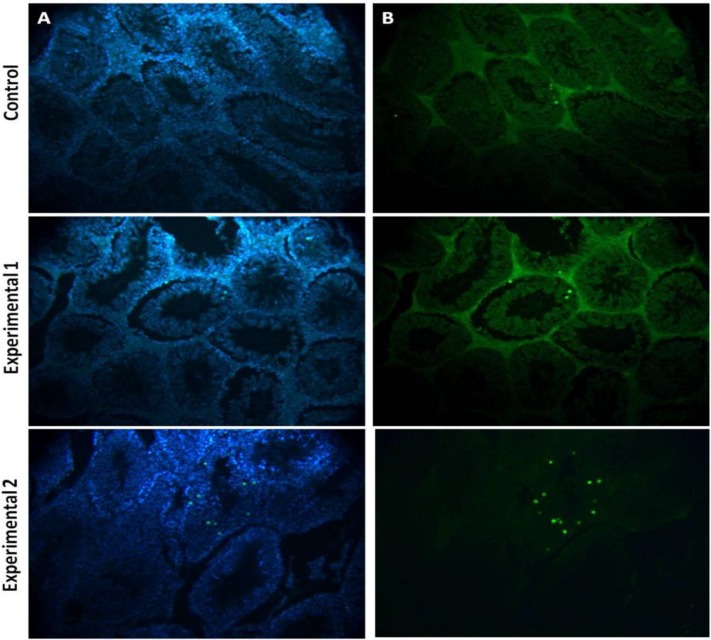
Effect of sevoflurane on the rate of TUNEL positive cells in the testis of mice. Nuclei stain by DAPI (A), Apoptotic cells (B). Magnification : ×200

The results revealed that the mean percent of apoptotic cells was 15±3%, 9±2 % and 5±1% in experimental groups 1, 2, and control (Figure 4).

**Table I:** Primers used for real-time PCR

**Genes**	**Primer Sequence**	**Gene bank code**
Bcl-2	FOR: 5΄- ACCGTCGTGACTTCGCAGAG-3΄	NM_00177410
	REV: 5΄- GGTGTGCAGATGCCGGTTCA-3΄	
Gapdh	FOR: 5′- GTG GAG TCA TAC TGG AAC ATG TAG-3΄	NM_008084
	REV: 5′- AAT GGT GAA GGT CGG TGT G-3΄	
Bax	FOR: 5΄- GCTGCAGACATGCTGTGGATC- 3΄	NM_007527
	REV: 5΄- TCACAGCCAGGAGAATCGCAC- 3΄	

**Table II:** Comparison of the sperm parameters in the control and experimental groups

**Groups**	**Viability (%)**	**Normal morphology (%)**	**Motility (%)**	**Count (×10** ^6^ **)**
Control	94.2+1.92*	69.6+6.38	63.4+5.68	10.902+0.12312
Experimental 1	53.6+5.899	46.4+5.89915*	21+17.1464	9.092+0.89895
Experimental 2	53.4+4.449	46.4+4.335*	38.8+9.3648*	10.036+0.2275

* Significant difference in comparison with the control group. Data presented as Mean±SE

*, **: significant difference in comparison with other groups in the same gene.

## Discussion

The present study proved that exposure to 2 MAC sevoflurane for 30 min/daily during 7 days had significant effects on the testicular tissue of mice. Frequent use of sevoflurane in children, in some congenital diseases, may have irreversible effects on fertility in male germ cells during the prepubertal period. The repeated use of the drug can be effective in the short term on effect the progenitor germ cell survival during spermatogenesis. 

Based on our results, integrity of tissue was intact in all groups, although spermatogonial cells population decreased in experimental group 2. Soubhia *et al* evaluated the effect of sevoflurane on transaminase on liver histology (23). Also, Elena *et al* studied some organs like spleen, kidney and liver after repeated sevoflurane anesthesia (24). Kaya *et al* revealed that testicular tissue and sperm parameters and sexual hormones were affected by chronic exposure to sevoflurane. Kaya et al used 1 MAC sevoflurane 2 h/day in 2 L/min oxygen for seven days and 1 MAC sevoflurane  in  2 L/min oxygen for 14 days, 2 h/day. 

Histological evaluation showed a significant difference in two experimental groups. Also, in Ceyhan’s study, effects of sevoflurane exposure on spermatogenesis and sperm parameters in rabbits was assessed. In Ceyhan et al study, rabbits received 20 exposure hr (4 hr/ 5 days) in concentrations 2.3% (1.2 MAC) sevoflurane and 1.3% (1.2 MAC) isoflurane. Their results showed that sperm parameters significantly decreased in two experimental groups, compared to the controls. Ceyhan *et al* showed chronic exposure to the sevoflurane had negative effects on the spermatogenesis and sperm morphology. Based on our results; 2 MAC sevoflurane in comparison with 1 MAC sevofleourane exposure leads to induction of apoptosis (25). Inhaled anesthetics had effect on human sperm motility according to Wang’s study. The sperm parameters were significantly improved after 0.5-4 hr exposure to isoflurane at the standard concentration and decreased slowly at high concentration dose (42%-84%) (26).

The effect of isoflurane on human sperm motility and vitality at the safe concentration was reversible when the anesthetic exposures were stopped. Sevoflurane had no effects on human sperm parameters at either the standard or high concentration. Leydig cells as the most susceptible cells affected by to some drugs affected by exposure to cell the anesthesia drugs. impairment of Leydig cells affects the spermatogenesis (27). The lumen of seminiferous tubules in mice exposed to 2 MAC sevoflurane had spermatozoa and spermatogenesis was fully established (including elongated spermatids). 

Degenerative changes in the germinal epithelium of rats were seen after chronic exposure to isoflurane (28). The results of sperm analysis in experimental group 2 revealed that exposure to 2 MAC sevoflurane during neonatal period led to decrease in all sperm parameter in comparison with the control group after puberty. Sperm viability and motility decreased significantly in male rats exposed to 2 MAC sevoflurane in comparison with the control group. However, no significant differences were found in sperm count between the exposed and control groups.

The results suggested that exposure to 2 MAC sevoflurane induced cell apoptosis and the alteration in the levels of expression of Bax and Bcl_2_ genes. There were significant differences in the expression of Bax and Bcl2 in experimental group 2 in comparison with the control group. The findings demonstrated that the ratio of Bax expression was elevated to 0.01279±0.01 and 0. 1252±0.001 in experimental group 1 after exposure to 1 and 2 MAC sevoflurane. The ratio of Bax/Bcl2 in experimental group 2 was 9.3657 in comparison with 1.00708 in experimental group 1. The expression of Bcl2 gene was significantly lower in the exposed group 2 than in the control group. This finding is in agreement with Bedirli *et al* findings which demonstrated down regulation of the Bcl2 in exposed group in comparison with the control group (29). 

The expression level of Bax in exposed mice was significantly higher than in the control group. The findings of the current study are consistent with those of Bedirli *et al* who found Bax up regulation in the brain of the rats in exposed group compared to the control group. Bcl2 family proteins were identified as inhibitors of apoptosis and these proteins may prevent Bax expression. Radical generation is inhibited by Bcl2. The decrease in the Bcl2 expression level may lead to increase in the level of reactive oxygen species (29). Bedirli *et al* showed that TNF-α, IL-1β, and myeloperoxidase levels were down regulated and antioxidant enzyme levels were elevated in the sevoflurane group in comparison with the control and isoflurane groups. Proapoptotic genes (Tnf, Tp53, and Tnfrsf10b) expression were decreased and antiapoptotic genes (Prok2, Bcl2l2, Aven and Bcl2) expression were increased with sevoflurane exposure compared to the isoflurane. Sevoflurane and isoflurane exposure decreased malondialdehyde and increased Bax compared to the control. Based on Bedirli study, sevoflurane and isoflurane preconditioning ameliorates inflammation, cerebral lipid peroxidation, and histological structure. Proapoptotic molecules down regulation and antiapoptotic molecules up regulation may be related to this effect (29).

Total numbers of germs cells was lower in the experimental group 2 with 2 MAC exposure of mice to sevoflurane than in other experimental and control groups. Sperm cells within the deferent duct were rarely observed in the experimental group 2. The lumen of seminiferous tubules in the experimental group 2 did not contain any spermatozoa. The number of dead sperm cells of mice in the experimental groups 1 and 2 significantly increased in comparison with the controls. all of the sperm parameters in the experimental group 2 were significantly lower than in the control group. Testicular sperm numbers in Ding et al findings significantly decreased in the isoflurane exposed rats than in the control group (28). The population of apoptotic cells increased after exposure to 2 MAC sevoflurane. Also, the expression level of Bax in this experimental group was higher than the other groups. The results of real time PCR and TUNEL assay indicated that sevoflurane exposure had negative effects on the expression of apoptosis regulatory genes. The ratio of Bax/Bcl2 in this group was 9.3657. The ratio of Bax/Bcl2 in the control group was 0.01229. The results of apoptosis demonstrated that the ratio of Bax/Bcl2 in 2 MAC sevoflurane exposed mice was significantly higher than in the control group.

## Conclusion

The results demonstrated that the lumen of seminiferous tubules contained spermatozoa and spermatogenesis was fully established. Poor interactions found between Sertoli cells and germ cells after exposure to 2 MAC sevoflurane. The sperm morphology was significantly different in the experimental group 2 compared to the control group. Sperm viability and motility was lower in the both experimental groups compared to the control group. This study showed that continuous exposure to 2 MAC sevoflurane during neonatal period may have detrimental effects on seminiferous tubules structure after puberty. The minimum Bcl-2 expression was found in mice with 2 MAC exposure to sevoflurane. Also, apoptotic cells population was higher than other groups in 2 MAC sevoflurane exposure.
